# Treatment fidelity in a pragmatic clinical trial of music therapy for premature infants and their parents: the LongSTEP study

**DOI:** 10.1186/s13063-022-06971-w

**Published:** 2023-03-03

**Authors:** Tora Söderström Gaden, Christian Gold, Jörg Assmus, Ingrid Kvestad, Andreas Størksen Stordal, Łucja Bieleninik, Claire Ghetti

**Affiliations:** 1 GAMUT – The Grieg Academy Music Therapy Research Centre, NORCE Norwegian Research Centre AS, Bergen, Norway; 2 Regional Centre for Child and Youth Mental Health and Child Welfare, NORCE Norwegian Research Centre AS, Bergen, Norway; 3 Department of Clinical and Health Psychology, University of Vienna, Vienna, Austria; 4 NORCE Energy, Norwegian Research Centre AS, Bergen, Norway; 5 Department of Mathematics, University of Bergen, Bergen, Norway; 6 Department of Clinical and Health Psychology, Faculty of Social Sciences, Institute of Psychology, University of Gdańsk, Gdańsk, Poland; 7 GAMUT – The Grieg Academy Music Therapy Research Centre, University of Bergen, Bergen, Norway

**Keywords:** Research methods, Fidelity, Treatment fidelity, Randomized controlled trial, Pragmatic trial, Multinational trial, Non-pharmacological interventions, Music therapy, Premature infant, Parent-infant bonding

## Abstract

**Background:**

Treatment fidelity (TF) refers to methodological strategies used to monitor and enhance the reliability and validity of interventions. We evaluated TF in a pragmatic RCT of music therapy (MT) for premature infants and their parents.

**Methods:**

Two hundred thirteen families from seven neonatal intensive care units (NICUs) were randomized to receive standard care, or standard care plus MT during hospitalization, and/or during a 6-month period post-discharge. Eleven music therapists delivered the intervention. Audio and video recordings from sessions representing approximately 10% of each therapists’ participants were evaluated by two external raters and the corresponding therapist using TF questionnaires designed for the study (treatment delivery (TD)). Parents evaluated their experience with MT at the 6-month assessment with a corresponding questionnaire (treatment receipt (TR)). All items as well as composite scores (mean scores across items) were Likert scales from 0 (completely disagree) to 6 (completely agree). A threshold for satisfactory TF scores (≥4) was used in the additional analysis of dichotomized items.

**Results:**

Internal consistency evaluated with Cronbach’s alpha was good for all TF questionnaires (*α* ≥ 0.70), except the external rater NICU questionnaire where it was slightly lower (*α* 0.66). Interrater reliability measured by intraclass correlation coefficient (ICC) was moderate (NICU 0.43 (CI 0.27, 0.58), post-discharge 0.57 (CI 0.39, 0.73)). Gwet’s AC for the dichotomized items varied between 0.32 (CI 0.10, 0.54) and 0.72 (CI 0.55, 0.89). Seventy-two NICU and 40 follow-up sessions with 39 participants were evaluated. Therapists’ mean (SD) TD composite score was 4.88 (0.92) in the NICU phase and 4.95 (1.05) in the post-discharge phase. TR was evaluated by 138 parents. The mean (SD) score across intervention conditions was 5.66 (0.50).

**Conclusions:**

TF questionnaires developed to assess MT in neonatal care showed good internal consistency and moderate interrater reliability. TF scores indicated that therapists across countries successfully implemented MT in accordance with the protocol. The high treatment receipt scores indicate that parents received the intervention as intended. Future research in this area should aim to improve the interrater reliability of TF measures by additional training of raters and improved operational definitions of items.

**Trial registration:**

Longitudinal Study of music Therapy’s Effectiveness for Premature infants and their caregivers – “LongSTEP”. ClinicalTrials.gov Identifier: NCT03564184. Registered on June 20, 2018

**Supplementary Information:**

The online version contains supplementary material available at 10.1186/s13063-022-06971-w.

## Background

Fidelity refers to the degree to which the delivery of an intervention adheres to the protocol or program developed. During the last decades, assessment of fidelity has had an increasing significance for evaluations, treatment effectiveness research, and service administration [1]. In multi-site studies, fidelity criteria are essential to ensure that interventions are conducted uniformly across sites, since this affects the reliability and validity of the intervention and conclusions about intervention effectiveness [ 2, 3]. A non-significant result might be the result of an ineffective intervention or could be due to interventionists not adhering to the protocol [1].

In clinical trials reporting on treatment interventions, the term treatment fidelity (TF) denotes the fidelity or integrity of an intervention [2]. Different methodological strategies can be applied to assess TF. Bellg et al. propose a five-component model for TF assessment in behavioural studies, including the design of the study, training of providers, treatment delivery, treatment receipt, and enactment of treatment skills [2]. These components are mutually exclusive and failing to attend to any of the components could compromise the internal validity of the study [3]. The first two components are considered in the development and preparation of a study, whereas the three last components can be assessed during and after the implementation of an intervention. Training of providers is a central part of enhancing TF with behavioural interventions, as it often requires learning new skills that might differ from clinicians’ existing training and experience [2]. Standardized training and monitoring and maintaining of provider skills are some of the recommendations of Bellg et al. [2] Treatment delivery (TD) refers to the extent the provider of treatment has adhered to the guidelines for the intervention and delivered treatment as intended. Treatment receipt (TR) refers to the degree to which the participant understands the treatment and their ability to perform protocol-related skills and strategies during the intervention [2]. Assessment of treatment enactment (TE) requires processes to monitor and improve the ability of patients to perform treatment-related strategies and skills in their daily lives between sessions or after the intervention period. In this article, we report on TD and TR assessed during the implementation of the Longitudinal Study of music Therapy’s Effectiveness for Premature infants and their caregivers—LongSTEP (ClinicalTrials.gov identifier NCT03564184) [4].

The intervention assessed in LongSTEP was a music therapy (MT) approach carried out in the context of neonatal intensive care units (NICUs) in different countries across Europe, the Middle East, and South America. With MT, we refer to “the informed use of music, facilitated by a trained music therapist within a therapeutic relationship, whereby engagement in musical processes serves as a resource to promote health” [5]. MT was first introduced to NICUs in the early 1990s [6, 7] with early research demonstrating positive effects on physiological and behavioural outcomes of premature infants, such as respiratory rate, oxygen saturation, heart rate, weight gain, and feeding patterns [8–11]. Within the last decade, MT in NICU has evolved in line with principles of family-centred care, supporting both infant development and parental well-being, including facilitating early parent-infant relationship through empowering parents in their parental roles and understanding of their infant [12–24]. We designed a pragmatic randomized controlled trial (RCT) [4] to evaluate longer-term parent-infant mutual outcomes, an identified gap in the knowledge base [25]. Since MT in NICU has predominately been conducted within the context of US health care [26, 27], we wished to contribute to knowledge development through collaboration with research partners from a broader range of cultural contexts where MT had not yet been systematically implemented in neonatal care, including Argentina, Colombia, Israel, Norway, and Poland. Given the international and multi-cultural nature of our trial, we were particularly interested in evaluating TF. Furthermore, to our knowledge, no clinical trials of MT in NICU have systematically evaluated TF. In the context of the LongSTEP trial, we developed and implemented strategies to enhance and assess TF, drawing upon experiences and examples from other MT trials with different populations [28–30].

The overall aim of this article is to report on TF in the LongSTEP trial, through evaluating the reliability of TF questionnaires developed for the trial, and the extent to which MT in the two intervention phases was a uniform intervention across therapists at the different research sites. Consistent with a pragmatic approach [24], intervention delivery included a balance of guiding principles for the intervention, combined with flexibility and openness to the clinicians’ interpretations and adaptions of the intervention to better fit usual care in their cultural context. Our research questions were as follows: (1) What are the internal consistency and interrater reliability of the TF questionnaires? (2) To what extent did the music therapists adhere to the essential elements of the intervention protocol? (3) To what extent did parents perceive MT to be in line with essential elements of the protocol?

## Methods

### Study design and participants

The participants were families with preterm infants born before 35 weeks gestational age (GA), likely to be hospitalized at least 2 weeks from inclusion, and declared by NICU staff as medically stable to start MT (typically after 26 weeks post-menstrual age) [4]. The included NICUs were level III and IV [31] units located in Argentina, Colombia, Israel, Norway, and Poland, all countries with high levels of parent presence in the NICU. Families were randomized to receive standard care, or standard care plus MT during NICU hospitalization, and/or during a 6-month follow-up period post-discharge. Participants in the control group were required not to receive any music-related interventions during the intervention period, and therapists were instructed to do MT sessions in individual patient rooms, if possible, to reduce contamination.

### Intervention

**Fig. 1:**
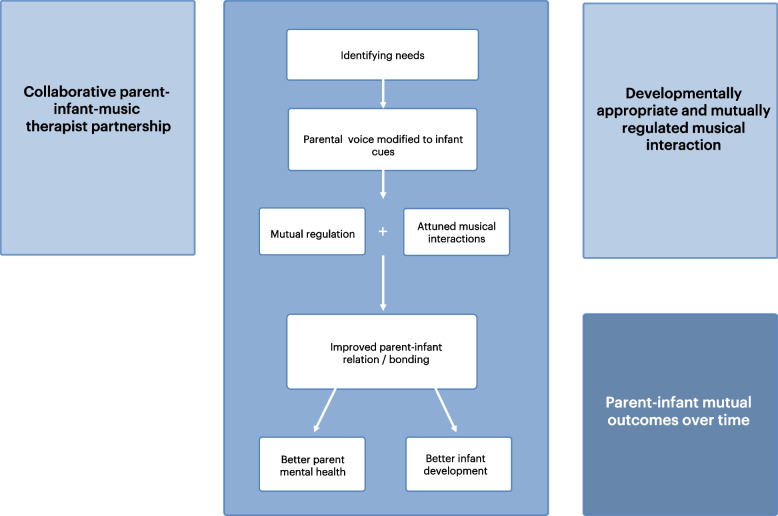
Key functions and proposed mechanisms of change

The MT intervention consisted of parent-led, infant-directed singing supported by a music therapist [5]. The singing was adapted in accordance with infant PMA and matched to the infant state and engagement/disengagement cues throughout the sessions. For infants aged ~ 26–32 weeks PMA, MT contained cautious use of (predominantly) parental singing and toned voice (e.g. single notes, simple melodies, or short musical phrases adapted from children’s songs or parent-preferred music) [ 5]. Our approach builds on previous models and approaches to MT in NICU [10, 17, 27, 32–35], with guiding principles founded in theories such as resource-oriented MT [36, 37], the mutual regulation model [38, 39], family-centred principles, and developmental care models [40, 41]. See further details in a separate article describing the theoretical foundation and intervention protocol [42]. Seven elements represent essential functions and processes that in combination can lead to therapeutic change. These elements should be present in each session regardless of the infant post-menstrual age or the phase in which MT is provided [42]. These were (1) observation and dialogue on infant’s needs prior to and during MT sessions, (2) dialogue with parents on their state and needs prior to sessions, (3) voice serves as the main instrument, (4) parental voice serves as the most prominent musical voice, (5) music therapist provides opportunities for parents to actively participate, (6) music is modified to infant cues and responses, and (7) parents’ culture and musical preferences and abilities are integrated into sessions (see Additional file 1). The key functions inherent in these elements are summarized in Fig. 1 to illustrate how the elements relate to each other and promote therapeutic change [43]. We want to emphasize that MT in routine clinical practice involves a high degree of individualization, flexibility, and improvization. We aimed for these elements to remain present by articulating guiding principles and essential elements rather than creating a detailed, rigorous intervention protocol. In the process of articulating guiding principles and elements, one challenge was to provide descriptions that were specific enough to enable consistent implementation across sites, without compromising the necessary adjustments each therapist had to do for their specific cultural context and settings, and for each family’s needs [43]. Per-protocol MT during NICU hospitalization comprised three weekly 20–30-min sessions throughout hospitalization of minimum 2 weeks (minimum 6 and maximum 27 sessions). Parents and infants participated in MT together and sessions were realized at bedside or in the family’s room during skin-to-skin-time, feeding, or with the infant lying in the incubator or cot. The number and average length of the session were tracked. Families randomized to MT during follow-up participated in seven monthly 45–60-min sessions over a 6-month period. Follow-up sessions were carried out at home, in the hospital, or at other health facilities. MT was adapted to the two phases in accordance with the intervention protocol [42].

### Treatment delivery

#### Training of providers

To monitor provider skills and delivery, all therapists submitted recordings of themselves carrying out sessions early in the implementation phase so that the core team could assess the need for additional training or support. The recordings used for this quality control purpose were excluded from the TF analysis. The music therapists were also encouraged to use a tracking form to increase awareness of aims, techniques, and progress across the course of the MT sessions. Supervision was another strategy to support the successful implementation and adherence to the guiding principles and essential elements of the MT. All therapists participated in online group supervision at least twice and online individual supervision at least once during the implementation period. The aim was to increase therapists’ self-awareness and to provide a space where challenges could be discussed openly, strategies could be shared, and experiences celebrated with peer support. These sessions also helped highlight how therapists were flexibly implementing the essential elements in alignment with specific cultural and context-based frames. Eleven music therapists were trained to deliver the MT intervention in our study. All eleven were female, masters-prepared music therapists, of which two were in the terminal stage of their degree. Six of the music therapists in the study participated in the in-person 1-day training consisting of lectures and practical exercises based on the intervention protocol during the study’s kick-off meeting. Five music therapists joined after study initiation and received online training sessions with the same content.

#### Treatment fidelity questionnaires

Five TF questionnaires were developed along with the theoretical foundation and intervention protocol [42], translating the intervention’s essential elements into items of behaviour we predicted would be audible or observable in the sessions (see Additional file 2). One element was not observable for all raters because of reliance on insight into the MT process and was therefore only included in the music therapist and parent questionnaires. Another element was only observable with video recordings and was included in the post-discharge tools only. The TF questionnaires were designed with Likert-scaled items (each 0–6), with anchors “I completely disagree” to “I completely agree”. A threshold of ≥4 per item was decided a priori as a satisfactory level of TF, with higher numbers indicating better therapist adherence and parent-perceived receipt of the item. Four TD questionnaires were created in accordance with (a) the phase within which MT was delivered, (b) which elements could feasibly be distinguished in audio versus video recordings of sessions, and (c) who was completing the rating. The questionnaires were *Treatment Delivery Questionnaire for Music Therapist Self-ratings, NICU* (seven items) *and post-discharge version* (eight items), and *Treatment Delivery Questionnaire for External Raters, NICU* (six items) *and post-discharge version* (seven items) (see Additional file 3). Each therapist’s sessions were reviewed by the corresponding therapist and two external raters who understood the language spoken and were educated in MT or psychology. Raters were provided with descriptions of behaviours related to each item to look for and were instructed to listen to or watch the recorded session once in its entirety, while filling out the questionnaire. The fifth questionnaire developed was the *Treatment Receipt Questionnaire* (nine items), where parents who received MT in one or both phases were instructed to think back on their experiences with MT as a whole and evaluate the degree to which they perceived the guiding principles of the MT intervention (see Additional file 4). The TD questionnaires were pilot tested by two members of the study core team and the TR questionnaire was discussed with the user advisory group who suggested simplifying the language. Changes were made accordingly before implementation.

### Data collection

Treatment delivery analysis was based on recordings from approximately 10% of each therapist’s participants, evaluated by the corresponding music therapist and two external raters per therapist. Sessions during NICU stay were audio recorded, and during follow-up were video recorded for all sites, except one that could not obtain permission to video record. Music therapists were responsible for audio/video recording their own sessions. Video instructions were to aim for a frame that showed both parent(s) and infant. Participants for TD analysis were randomly selected using www.randomresult.com with the “Pick items” function. If the material from the selected participant was not possible to use (e.g. missing video/audio, participant dropped out of the study), a new participant was drawn randomly. We strived to evaluate sessions that were distributed over time, avoiding the first and last sessions as the first sessions were used to explain and demonstrate aspects of the intervention and the last sessions to sum up content from the course of MT and dialogue about continued, independent use of music. Hence, we expected that the first and last sessions would include minimal levels of interaction between parents and infants or singing. When participants received per-protocol MT during the NICU phase, we analysed recordings of sessions 3, 5, and 7. When participants had fewer than seven sessions, we analysed sessions 2, 4, and 6. If a participant received more than 10 sessions, the 11th was added with the intention of investigating drifting; however, we did not have a sufficient sample for this analysis. Recordings of two sessions per participant were evaluated from the follow-up phase, either three and six, three and five, or four and six, based on the useable video. The variation of session numbers in the final data material was due to missing recordings. Parent self-report ratings at the 6-month assessment served as data for the analysis of TR.

### Analysis

Descriptive methods were applied to characterize the two participant samples for TD and TR. Categorical data were analysed with frequency and percentage, and numerical data with mean, standard deviation, and range due to normally distributed data. The internal consistency of each TF questionnaire was evaluated with Cronbach’s alpha [44] with alphas of ≥0.70 indicating good internal consistency [45]. Interrater reliability (IRR) between music therapists and external raters was evaluated per item and composite score with intraclass correlation coefficient (ICC) with a two-way model, single measurement, and absolute agreement. Additionally, we calculated the agreement of categorical items dichotomized to above/below threshold of satisfactory adherence (≥4). Because of the high prevalence of single-item alternatives for some items, we used Gwet’s AC [46] instead of kappa, due to the known weaknesses of kappa in this case [47]. Mean TD scores per item, therapist, and composite score (mean scores across external ratings and music therapist self-ratings) were calculated from ratings from the two intervention phases (NICU and post-discharge). Mean TR scores per item and composite score per participant and composite score across intervention conditions (MT in NICU, post-discharge, or both) were calculated from parent ratings. For these analyses, it was not necessary to consider who was the first and who was the second external rater. Statistical analyses were done with software R version 4.1.0 [48] and graphics with Matlab 2021b [49].

## Results

In total, 72 NICU and 40 post-discharge sessions of 39 unique participants (Table 1) were rated by 10 music therapists and 13 external raters for TD assessment. For post-discharge sessions, we also reviewed video characteristics of who was present in sessions and their visibility in the recordings for data quality purposes. Mothers were present in all sessions, fathers in 37.5%, siblings in 41%, and grandmothers in 15% of the sessions. Mothers were fully visible in 81% of the videos and fathers in 83% of the sessions they attended, while the infants were fully visible in only 53% of the videos. The same applied to music therapists who were visible in 53% of the videos. Treatment receipt was evaluated by 135 parents at the 6-month assessment (Table 2).

**Table 1:** Sample characteristics of treatment delivery

	NICU MT		PD MT		MT both phases	
	*N*	Value	*N*	Value	*N*	Value
Infant female sex, no. (%)	27	10 (37%)	30	12 (40%)	18	7 (39%)
Infant birth weight, mean grammes, (SD)	27	1355 (405)	30	1365 (401)	18	1314 (358)
Infant GA at birth, mean weeks, (SD)	27	30.1(2.5)	30	30.0 (2.6)	18	29.6 (2.4)
Mother age, mean years (SD)	26	31.8 (4.0)	29	32.0 (5.2)	17	32.3 (3.9)
Mother education, mean years (SD)	27	15.8 (3.8)	30	16.1 (3.1)	18	16.4 (2.7)
Mother usual work situation, no. (%)	27	-	30	-	18	-
Full-time- or self-employed		19 (70%)		21 (70%)		12 (67%)
Other^a^		8 (30%)		9 (30%)		6 (33%)
Mother civil status, no. (%)	26	-	30	-	18	-
Married		19 (73%)		20 (67%)		13 (72%)
Living together but not married		7 (27%)		9 (30%)		5 (28%)
Others		-		1 (3%)		-
Father age, mean years (SD)	27	34.5 (4.4)	30	34.2 (5.3)	18	34.2 (5.0)
Father education, mean years (SD)	26	15.1 (3.6)	29	15.0 (3.4)	17	15.6 (3.2)
Father usual work situation, no. (%)	27	-	30	-	18	-
Full-time- or self-employed		26 (96%)		29 (97%)		17 (94%)
Other^a^		1 (4%)		1 (3%)		1 (6%)

*Note: *One participant was removed from the sample because of language challenges between the therapist and family

*Abbreviations*: *NICU* neonatal intensive care unit, *MT* music therapy, *PD* post-discharge, *SD* standard deviation, *GA* gestational age

^a^Other includes part-time, homemaker/stay-at-home parent, student, unemployed and seeking work, and unemployed due to ill health or a disability

**Table 2:** Sample characteristics of treatment receipt

	NICU MT		PD MT		MT both phases	
	*N*	Value	*N*	Value	*N*	Value
Infant female sex, no. (%)	49	22 (45%)	41	23 (56%)	45	21 (47%)
Infant birth weight, mean grammes, (SD)	49	1344 (440)	41	1423 (430)	45	1415 (414)
Infant GA at birth, mean weeks, (SD)	49	30.2 (2.7)	41	32.85 (5.47)	45	30.34 (2.57)
Mother age, mean years (SD)	49	31.9 (5.6)	40	33.07 (2.21)	44	34.07 (5.20)
Mother education, mean years (SD)	48	15.2 (3.6)	39	16.03 (2.97)	44	16.36 (2.80)
Mother usual work situation, no. (%)	49	-	41	-	45	-
Full-time- or self-employed		30 (61%)		33 (80.5%)		33 (73%)
Other^a^		19 (39%)		8 (19.5%)		12 (27%)
Mother civil status, no. (%)	48	-	41	-	45	-
Single		3 (6%)		4 (10%)		2 (4%)
Married		33 (69%)		25 (61%)		32 (71%)
Living together but not married		12 (24%)		10 (24%)		11 (24%)
Others		-		2 (5%)		-
Father age, mean years (SD)	47	35.1 (6.8)	39	35.4 (5.7)	42	36.4 (5.3)
Father education, mean years (SD)	45	14.3 (4.0)	38	15.6 (3.4)	41	15.7 (7.2)
Father usual work situation, no. (%)	47	-	39	-	42	-
Full-time- or self-employed		44 (94%)		38 (97%)		39 (93%)
Other^a^		3 (6%)		1 (3%)		3 (7%)

*Note: *One participant was removed from the sample because of language challenges between the therapist and family

*Abbreviations*: *NICU* neonatal intensive care unit, *MT* music therapy, *PD* post-discharge, *SD* standard deviation, *GA* gestational age

^a^Other includes part-time, homemaker/stay-at-home parent, student, unemployed and seeking work, and unemployed due to ill health or a disability

### Reliability of treatment fidelity questionnaires

We conducted reliability analyses of the questionnaires, assessing internal consistency and interrater reliability. Internal consistency of the scales was measured with Cronbach’s alpha indicating good internal consistency (≥0.70) for all except the NICU external rater questionnaire which scored slightly lower (*α* (CI) 0.66 (0.60, 0.73), Table 3). For all questionnaires, most items appeared to be worthy of retention resulting in a decrease in or no change in alpha if removed (see Additional file 5). Based on these alpha calculations, it was decided to keep all items in all scales and to calculate composite scores as planned.

**Table 3:** Cronbach’s alpha for treatment fidelity questionnaires

	Treatment delivery		Treatment receipt
	NICU	Post-discharge	Both phases
Music therapist self-rater questionnaire	0.75 (0.6 7, 0.82)	0.87 (0.84, 0.91)	-
External rater questionnaire	0.66 (0.60, 0.73)	0.80 (0.76, 0.84)	-
Parent questionnaire	-	-	0.79 (0.74, 0.83)

*Note: *All values are raw *α* (CI)

*Abbreviations*: *CI* confidence interval, *NICU* neonatal intensive care unit

**Fig. 2:**
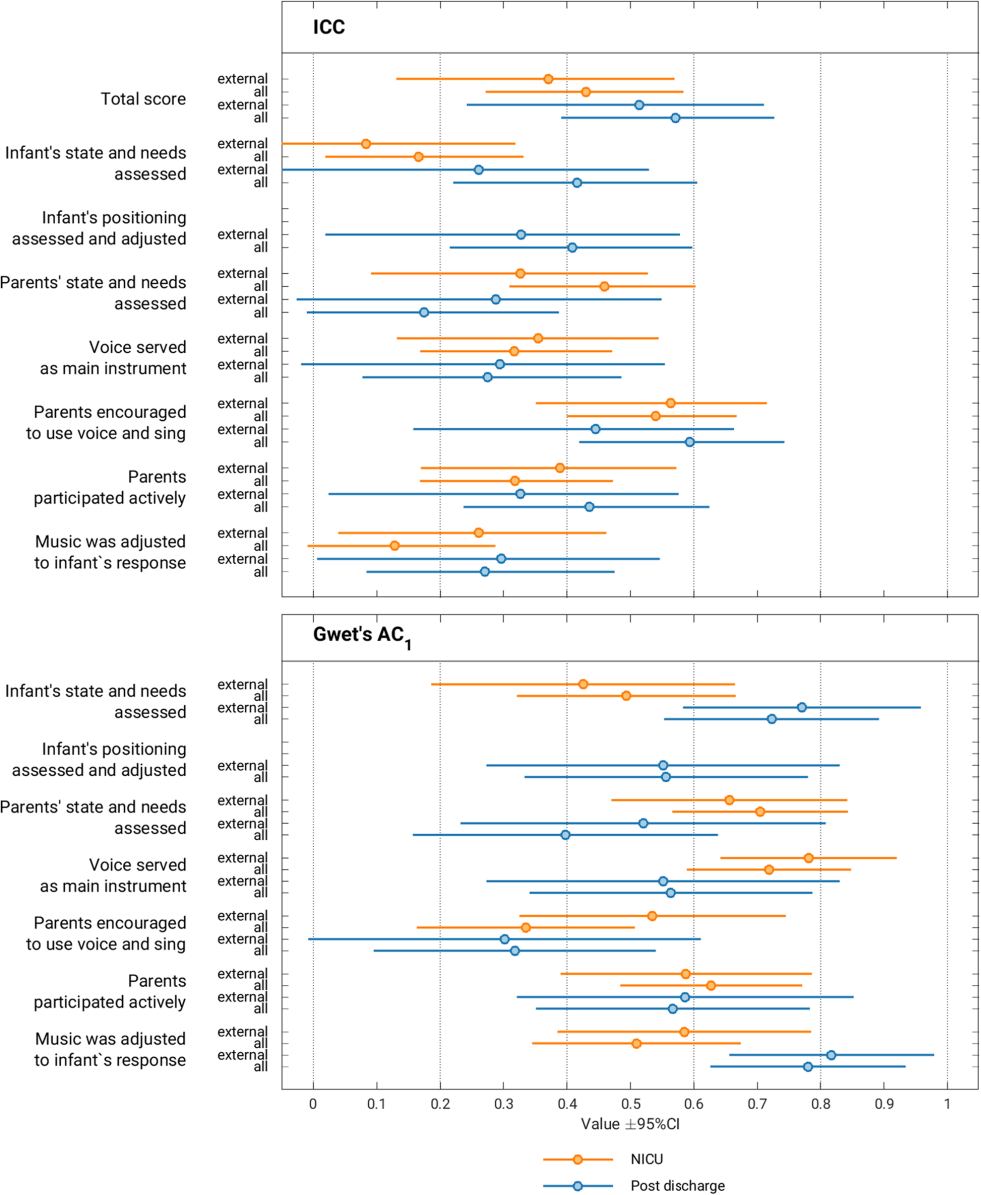
Interrater reliability of treatment delivery questionnaires

Interrater reliability (IRR) of the TD composite scores across music therapist self-rater and external rater versions was moderate with ICC 0.43 (CI 0.27, 0.58) (Fig. 2). Gwet’s AC for the dichotomized items varied between 0.32 (CI 0.10, 0.54) and 0.72 (CI 0.55, 0.89) (Fig. 2).

### Treatment delivery and treatment receipt

**Fig. 3:**
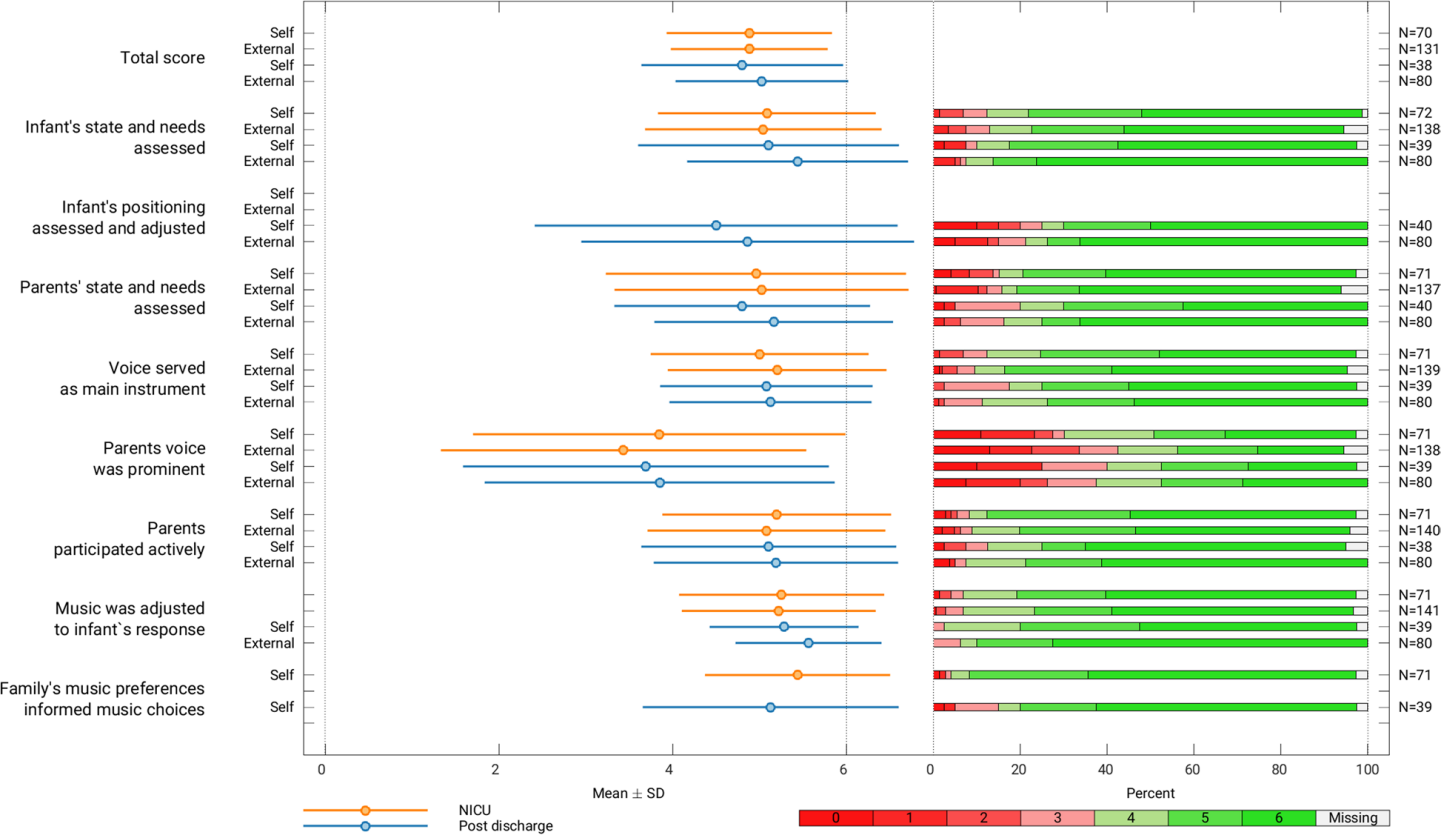
Treatment delivery scores per item

**Fig. 4:**
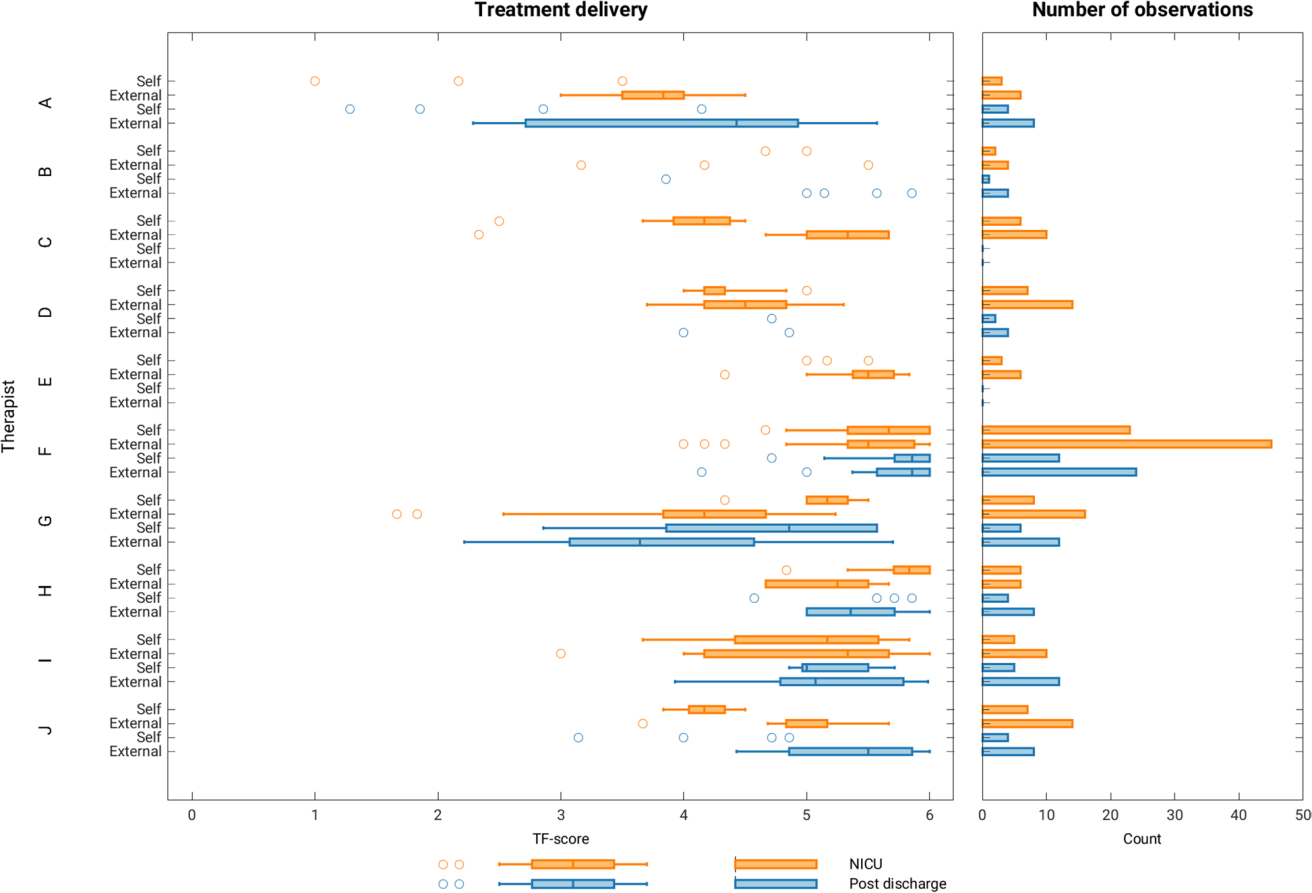
Treatment delivery scores per therapist

**Fig. 5:**
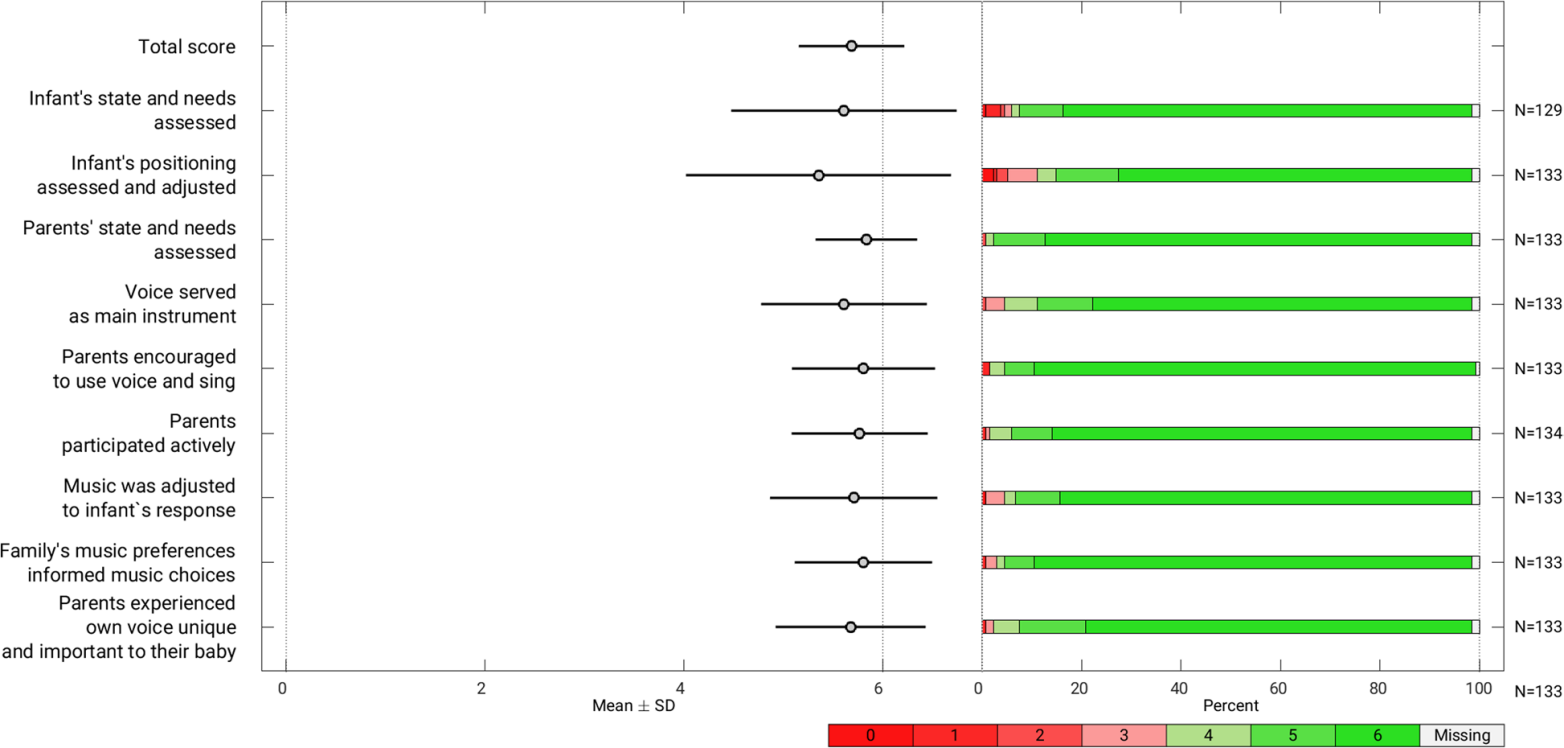
Treatment receipt scores per item

The mean composite TD score across raters for NICU sessions was 4.88 (0.92) and 4.95 (1.05) for post-discharge sessions, scoring between 1 and 2 Likert points away from “I completely agree” (Fig. 3). The mean TD composite score per therapist ranged from 3.17 to 5.46 for NICU phase and 3.51 to 5.65 post-discharge (Fig. 4). Treatment receipt mean scores were very high with the NICU group mean (SD) of 5.66 (0.50), post-discharge group 5.65 (0.71), and 5.71 (0.40) for the group who received MT in both phases. The mean (SD) TR composite score across groups was 5.68 (0.53) (Fig. 5).

## Discussion

We reported on treatment fidelity in a multi-national clinical trial, LongSTEP. Average TD composite scores indicate that music therapists adhered to central elements of the intervention protocol to a satisfactory degree and that MT was a uniform intervention during the NICU stay and follow-up post-discharge. TR scores were also satisfactory, with several items scoring very high, suggesting that parents received the essential elements of the intervention. Parents who received MT during both NICU and post-discharge had the highest TR composite score, but differences between receiving MT in one or both phases were smaller than expected. Due to cultural differences between the participating countries, variation in experience of therapists and raters, and the complex nature of the intervention, we were pleasantly surprised by these results. In line with a pragmatic approach [24], we were seemingly successful in our implementation of TF strategies, including the provision of guidelines for intervention delivery that left sufficient room for flexibility and individual tailoring required to fit each site’s usual care across a range of cultural contexts. This indicates a high degree of clinical applicability of our MT approach outside the research context.

Treatment fidelity questionnaires had acceptable internal consistency and moderate interrater reliability (IRR). The moderate results on IRR could be due to the complex, flexible character of the intervention. Interpreting musical interaction and subtle infant behaviours from recordings with varying quality is challenging. We also believe that rater training could have been better. Whereas the music therapists received standardized training in the intervention and supervision, the external raters received written instructions for evaluation of the sessions and written individual support when they requested it. We recommend providing more systematic training of raters, including establishing adequate interrater reliability with the intervention trainer using sample videos before commencing rating of study data. We also recommend recruiting raters who have a similar level of familiarity with the intervention and population in question, since the level of experience likely influences assessments. We did not complete an analysis of test-rest reliability due to a lack of resources but recommend such analysis. Instructions for raters should also include a narrow time window for completing ratings and we suggest that ratings be completed shortly after sessions take place, so that potential problems with recordings or other factors are discovered early in the process.

Overall TD scores were high but one item concerning parents’ voices serving as prominent musical voices during MT, which scored below the threshold for satisfactory adherence (≥4). This finding could be explained by the fact that singing to one’s baby is an intimate action that many parents can feel shy or insecure about with others present. At some sites, lack of space meant that several families shared rooms, which poses several challenges including the risk of contamination. Control group families were asked to avoid participating in any music-related intervention during the intervention period, but due to open-bay units and lack of space, intervention and control group families may at times have been in the same room. While MT was tailored individually, such that other families in the room would not have received MT per protocol, there is still a chance that they overheard tips and strategies and applied these independently. A cluster randomized design could have reduced this risk for contamination but was not chosen as it among other things would have required recruitment of more participants [50, 51]. We did however screen for contamination in the discharge assessment asking whether participants had learned from other parents in the NICU about using music with their baby. Out of the 99 standard care group participants who answered the item, only eight of them responded “yes”, which indicates that contamination was likely not a major issue. Having other families nearby might also have compromised the opportunity to provide a comfortable atmosphere where parents felt safe to sing and try out new things. During supervision, several music therapists reported addressing such challenges by encouraging parents to sing while still making sure they felt comfortable and respecting their reservations and needs. A feasibility study testing our intervention found that the use of the guitar was effective to support mothers’ musical engagement, allowing them to feel more confident when singing [52].

It may be that expectations regarding active participation through the use of voice vary considerably among external raters, music therapists, and parents. Where external raters and music therapists might have expected that parents would sing often in most sessions, and hence rated this item low when singing occurred less often than expected, parents might have felt that any amount of singing was more than they would have done without MT and thus perceived their own vocal engagement as substantial. An item unique to the TR questionnaire addressed whether parents experienced their voices as being unique and important to their baby. This item had a very high (>5) score, which suggests that parents experienced their own voices as unique resources, despite the music therapist and external raters rating parents low on the use of voice in sessions.

While the results from the main timepoint of the LongSTEP trial are not yet published, results from the preliminary timepoint of discharge report a non-significant effect of MT on mother-infant bonding, maternal depression, or parental anxiety [5]. Since our present analysis shows satisfactory levels of TF, these non-significant results do not seem to be the result of inconsistent implementation of the intervention but may rather indicate that the intervention was not well-matched for the specific outcome measures chosen. Parents’ TR scores suggest that the intervention did contribute to parents perceiving that they have something unique to offer their baby through using their voices. Through participation in MT, they also perceived essential elements about how music was adjusted to their baby’s needs in the moment, which benefitted them as transferable skills they could use on their own in their everyday lives—both between sessions during NICU hospitalization and follow-up and after the intervention period ended.

Our TF analysis has limitations. For TD evaluation, all raters knew when in the therapeutic process the session happened which might have affected raters’ expectations and the outcomes of the ratings. There were large differences between the therapists’ number of participants and sessions and hence large variation in the data from which the scores were calculated. It may be that the sample of participants for TD was not representative due to our strategy of excluding participants with missing video/audio. It is also possible that poor audio/video quality in some instances made certain behaviours correspondent with the intervention’s essential elements very difficult to observe. The TF questionnaires lacked an option for raters to report if the item was not possible to observe, and the degree to which raters reported poor data quality may have varied. In contrast to recordings strategically selected for TD assessment, parents who evaluated TR rated their overall experience with MT thinking back on the course of sessions over time, meaning they could base their evaluation on more sessions and probably a broader range of experiences.

LongSTEP was designed as a pragmatic trial aiming to increase the applicability of study results to real-world settings and usual treatment. However, through developing and implementing strategies to enhance, monitor, and evaluate TF which included the development of intervention guidelines [43], and monitoring and supervision of music therapists during the intervention period, one could argue that we actually moved slightly towards the explanatory end of the explanatory-pragmatic continuum [24].

## Conclusion

Treatment fidelity questionnaires developed to assess treatment delivery and treatment receipt of MT for premature infants and their parents in the LongSTEP study showed good internal consistency and moderate interrater reliability. Treatment delivery scores indicated that music therapists across a wide range of cultural contexts were able to successfully implement the complex behavioural intervention of our MT approach, adhering to the essential elements of the intervention protocol. This indicates the high clinical applicability of the LongSTEP approach to MT in NICU. Parents’ high treatment receipt scores support this notion and indicate specific areas where the intervention benefitted them above and beyond the LongSTEP trial’s primary and secondary outcomes. Parents experienced their own voices as unique resources in relation to their baby and likely developed skills transferable to their daily lives [53]. Future research in this area should aim to improve the interrater reliability of TF measures, for example by additional training and follow-up for raters and/or by improved operational definitions of items.

## Supplementary Information

**Additional file 1.** Essential elements of the LongSTEP approach to MT in NICU.

**Additional file 2.** Wording of items in treatment fidelity questionnaires.

**Additional file 3.** LongSTEP Treatment Delivery Tools.

**Additional file 4.** LongSTEP Treatment Receipt Questionnaire.

**Additional file 5: Table 4.** Reliability for LongSTEP treatment fidelity questionnaires if an item is dropped.

## Data Availability

The datasets used and analysed for this study are available from the corresponding author upon reasonable request.

## Abbreviations

CI: Confidence interval
DC: Discharge
GA: Gestational age
ICC: Intraclass correlation coefficient
IRR: Interrater reliability
M: Mean
MD: Median
MT: Music therapy
NICU: Neonatal intensive care unit
PD: Post-discharge
RCT: Randomized controlled trial
SC: Standard care
SD: Standard deviation
TD: Treatment delivery
TF: Treatment fidelity
TR: Treatment receipt
