# Supplementary material for: Treatment fidelity in a pragmatic clinical trial of music therapy for premature infants and their parents: the LongSTEP study
Source: Trials. 2023 Mar 3;24:160. doi: 10.1186/s13063-022-06971-w (PMC9983212; doi:10.1186/s13063-022-06971-w)
Supplement: Supplementary file 1 — Additional file 1. Essential elements of the LongSTEP approach to MT in NICU. [file 13063_2022_6971_MOESM1_ESM.docx]

**Appendix 1: Essential elements of the LongSTEP approach to MT in NICU**

1. *Observation and dialogue on infant’s needs prior to and during MT sessions*

This element relates back to principles of developmentally appropriate care for premature infants ^1, 2^ and aims to ensure that each session’s content is tailored to infant development, as well as their state and needs in the moment. ^3^ Each session should begin with an initial observation together with parents of the infant’s state and readiness for interaction. Physiological cues such as respiratory rate and pulse, skin colour, and behavioural cues such as motor activity and quality of movements, sleep/awake states, and responsivity help music therapist and parents to identify infant state and needs in the moment. Music therapist should articulate own observations along the way and encourage parents to do the same to promote dialogue, participation, and a shared understanding of the infant. Infant physical positioning should be observed and adjusted to support infant readiness and parent-infant mutual regulation.

1. *Dialogue with parents on their state and needs prior to session*

Preterm birth not only affects the infant but also parents and other family members. Parents of premature infants are at risk for significant stress ^4^, post-traumatic stress disorder ^5^, anxiety ^6^, and depression, which in turn might negatively affect parent-infant interaction. ^7^ Therefore, it is important to also check in with parents. A stressed or anxious parent might not be ready to engage in musical interaction without having a chance to express what they are experiencing and feeling. Parents should be provided time and space to express feelings and concerns, as well as needs they themselves wish the music therapist can meet.

1. *Voice serves as the main instrument*

The human voice is a contrast to the highly technological and often noisy environment of a NICU. In comparison to other musical instruments that have been used in NICU settings (e.g., harp, monochord, guitar) ^8-10^ the voice is also highly available, easy to adjust in terms of volume and intensity, and as such provides an accessible resource for both therapist, parents, and others who interact with the infant. Using the voice also affects breathing patterns, which provides a potential pathway to actively work with regulating stress, emotions, and anxiety in parents. ^11^

1. *Parental voice serves as the most prominent musical voice*

The voices most relevant and powerful for the infant are the parents’ own voices. The foetus develops the capacity for hearing during the second trimester meaning that sound is part of their sensory world long before they are born. ^12^ Parents’ voices, and particularly the pregnant mother’s voice, are therefore familiar to new-borns ^13, 14^ and may provide premature infants a sense of security and a link back to the safe and predictable environment of the womb. ^15^ Focusing on parent voice also relates back to principles of resource-oriented music therapy where personal resources and empowerment are central. ^16^

1. *Music therapist provides opportunities for parents to actively participate*

In line with principles of family-centred care we propose that parents should be supported to participate actively and eventually take on a leading role in the music therapy. Parents’ unique insight and knowledge about their baby is a resource in this parent-infant relational work, and the music therapist brings musical competence to this collaborative therapeutic relationship. To facilitate parent musical engagement, the music therapist should accommodate parents’ levels of musical experience and modify music accordingly (e.g., select comfortable vocal range). Parents should be supported in identifying and expanding their musical resources and through this learn to interpret and respond to the infant’s subtle signals in a new manner which is transferable to daily life settings when the music therapist is not present.

1. *Music is modified to infant cues and responses*

We believe premature infants possess potential to be communicative partners with their parents through their communicative musicality. ^17^. Although their communication cues are often subtle, and they cannot yet make their own music, premature infants can be active participants in MT through affecting and informing the song and interplay provided by the person singing. ^18^. However, premature infants are easily overstimulated which can be harmful to their development. ^19^ Therefore, it is crucial that all interaction is tailored to infants’ states and responses along the way. As in the initial observation, infant state and behaviour should be carefully monitored and impact the way musical interaction is carried out throughout the session, following guidelines for safe uses of music with premature infants expanding musical complexity and interaction progressively in line with infant GA and readiness ^10, 20, 21^.

1. *Parents’ culture and musical preferences and abilities are integrated into sessions*

In the same way that parents’ voices are the most relevant ones, parents’ own music preferences, culture, and musicality are a crucial part of the intervention, and should be integrated into the sessions. Parents are invited to share their favourite songs, or songs that might be particularly meaningful to them in the situation they are in, and the therapist demonstrates how these can be adapted in an infant-appropriate way. This is in line with Loewy’s concept “song of kin” ^8^ which includes everything from lullabies, parent-preferred popular music, and music that is representative of the family’s nationality, religion, or cultural background. Because our study includes five different countries, we emphasize the importance of the music therapists’ ability to sensitively perceive and respond to complex layers of cultures, contexts, and experiences of the families. ^22^

**References**

1. Als H. Newborn individualized developmental care and assessment program (NIDCAP): new frontier for neonatal and perinatal medicine. J Neonatal Perinatal Me 2009; 2: 135-147.

2. Rauh VA, Achenbach TM, Nurcombe B, et al. Minimizing adverse effects of low birthweight: Four-year results of an early intervention program. Child Dev 1988: 544-553.

3. Gaden TS, Ghetti C, Kvestad I, et al. The LongSTEP approach: Theoretical framework and intervention protocol for using parent-driven infant-directed singing as resource-oriented music therapy. Nord J Music Ther 2021: 1-26.

4. Sloan K, Rowe J and Jones L. Stress and coping in fathers following the birth of a preterm infant. *Neonatal Nurs* 2008; 14: 108-115.

5. Winter L, Colditz PB, Sanders MR, et al. Depression, posttraumatic stress and relationship distress in parents of very preterm infants. *Arch Womens Ment Health* 2018; 21: 445-451.

6. Trumello C, Candelori C, Cofini M, et al. Mothers' depression, anxiety, and mental representations after preterm birth: A study during the infant's hospitalization in a neonatal intensive care unit. *Public Health* *Front* 2018; 6: 359.

7. Beeghly, M., Fuertes, M., Liu, C. H., Delonis, M. S., & Tronick, E. Maternal sensitivity in dyadic context: Mutual regulation, meaning-making, and reparation. In D. W. Davis & M. C. Logsdon (eds.) *Maternal sensitivity: A scientific foundation for practice.* Hauppauge, NY: Nova Science Publishers 2011, pp.45-69.

8. Loewy J. NICU music therapy: song of kin as critical lullaby in research and practice. Ann N Y Acad Sci 2015; 1337: 178-185.

9. Haslbeck FB and Bassler D. Clinical Practice Protocol of Creative Music Therapy for Preterm Infants and Their Parents in the Neonatal Intensive Care Unit. *J Vis Exp* 2020: e60412.

10. Nöcker-Ribaupierre M. Premature infants. In Bradt J (ed) *Guidelines for music therapy practice in pediatric care.* Gilsum, NH*:* Barcelona Publishers 2013, pp.66-104.

11. Gick ML. Singing, health and well-being: A health psychologist’s review. *Psychomusicology* 2011; 21: 176-207.

12. Graven SN and Browne JV. Auditory development in the fetus and infant. *Newborn Infant Nurs Rev* 2008; 8: 187-193.

13. Moon C. The role of early auditory development in attachment and communication. *Clin Perinatol* 2011; 38: 657-669.

14. DeCasper AJ and Fifer WP. Of human bonding: Newborns prefer their mothers' voices. *Science* 1980; 208: 1174-1176.

15. Loewy J, Stewart K, Dassler AM, et al. The effects of music therapy on vital signs, feeding, and sleep in premature infants. *Pediatrics* 2013; 131: 902-918.

16. Rolvsjord R. *Resource-oriented music therapy in mental health care*. Princeton, NJ: Citeseer, 2010.

17. Malloch S and Trevarthen C. *Communicative musicality: Exploring the basis of human companionship*. Oxford University Press, USA, 2009.

18. Shoemark H. Contingent Singing. *Voicework in Music Therapy: Research and Practice* 2011: 231.

19. Liu L, Johnson HL, Cousens S, et al. Global, regional, and national causes of child mortality: an updated systematic analysis for 2010 with time trends since 2000. *Lancet* 2012; 379: 2151–2161.

20. Hanson-Abromeit D. The Newborn Individualized Developmental Care and Assessment Program (NIDCAP) as a model for clinical music therapy interventions with premature infants. *Music therapy perspectives* 2003; 21: 60-68.

21. Standley JM, Walworth D and American Music Therapy A. *Music therapy with premature infants: research and developmental interventions*. Silver Spring, MD: American Music Therapy Association, 2010.

22. Shoemark H and Ettenberger M. *Music therapy in neonatal intensive care: Influences of culture*. 2020.
