# Supplementary material for: Treatment fidelity in a pragmatic clinical trial of music therapy for premature infants and their parents: the LongSTEP study
Source: Trials. 2023 Mar 3;24:160. doi: 10.1186/s13063-022-06971-w (PMC9983212; doi:10.1186/s13063-022-06971-w)
Supplement: Supplementary file 2 — Additional file 2. Wording of items in treatment fidelity questionnaires. [file 13063_2022_6971_MOESM2_ESM.docx]

**Appendix 2: Wording of items in treatment fidelity questionnaires**

*Music therapist self-ratings, NICU*

1. In the beginning of the session, music therapist and parent(s) observe and dialogue on infant’s current state and needs prior to presenting any music
2. In the beginning of the session (and during, as appropriate), music therapist dialogues with parent(s) about parent’s own state, needs, and concerns
3. Voice serves as the main instrument during the music therapy session
4. Parental voice serves as a prominent musical voice during the music therapy session
5. Music therapist provides opportunities for parents to actively participate during the music therapy session
6. Music is modified in response to infant’s cues and responses during the music therapy session
7. Parents’ musical preferences and abilities are integrated and accommodated into the music therapy session

*Music therapist self-ratings, post-discharge*

1. In the beginning of the session, music therapist and parent(s) observe and dialogue on infant’s current state and needs prior to presenting any music
2. In the beginning of the session (and during, as appropriate), music therapist dialogues with parent(s) about parent’s own state, needs, and concerns
3. In the beginning of the session (and during, as appropriate), infant’s physical positioning is attended to and adjusted to support infant self-regulation and enhance conditions for parent--infant interaction
4. Voice serves as the main instrument during the music therapy session
5. Parental voice serves as a prominent musical voice during the music therapy session
6. Music therapist provides opportunities for parents to actively participate during the music therapy session
7. Music is modified in response to infant’s cues and responses during the music therapy session
8. Parents’ musical preferences and abilities are integrated and accommodated into the music therapy session

*External raters, NICU*

1. In the beginning of the session, music therapist and parent(s) observe and dialogue on infant’s current state and needs prior to presenting any music
2. In the beginning of the session (and during, as appropriate), music therapist dialogues with parent(s) about parent’s own state, needs, and concerns
3. Voice serves as the main instrument during the music therapy session
4. Parental voice serves as a prominent musical voice during the music therapy session
5. Music therapist provides opportunities for parents to actively participate during the music therapy session
6. Music is modified in response to infant’s cues and responses during the music therapy session

*External raters post-discharge*

1. In the beginning of the session, music therapist and parent(s) observe and dialogue on infant’s current state and needs prior to presenting any music
2. In the beginning of the session (and during, as appropriate), music therapist dialogues with parent(s) about parent’s own state, needs, and concerns
3. In the beginning of the session (and during, as appropriate), infant’s physical positioning is attended to and adjusted to support infant self-regulation and enhance conditions for parent--infant interaction
4. Voice serves as the main instrument during the music therapy session
5. Parental voice serves as a prominent musical voice during the music therapy session
6. Music therapist provides opportunities for parents to actively participate during the music therapy session
7. Music is modified in response to infant’s cues and responses during the music therapy session

*Parent ratings of treatment receipt*

1. Before the music started, the music therapist and I looked at my baby and discussed my baby’s current state (for example asleep, awake, calm, distressed), and what my baby needed in the moment
2. Before the music started, the music therapist and I checked if my baby was laying (or sitting) in a way that helped my baby be ready for music, and if needed we adjusted the baby’s position
3. Before the music started, the music therapist asked me about how I was feeling, and whether I had any particular concerns, as well as any wishes or needs for the music therapy session
4. We used singing and our voices more than any other instrument during music therapy sessions
5. The music therapist encouraged me to use my voice and singing to connect with my baby in the music therapy sessions
6. I actively participated in the music therapy sessions. For example, by singing, humming, touching and/or moving my baby to the music, and/or making up songs and lyrics
7. The music was adjusted according to my baby’s reaction to it. For example, we changed tempo, volume, or melody to interact with my baby, or paused and made the music simpler if the baby seemed to need a break
8. We used music that my family and I like in the music therapy sessions
9. I experienced in the music therapy sessions that I have something unique and important to offer my baby through my voice
