# Supplementary material for: Treatment fidelity in a pragmatic clinical trial of music therapy for premature infants and their parents: the LongSTEP study
Source: Trials. 2023 Mar 3;24:160. doi: 10.1186/s13063-022-06971-w (PMC9983212; doi:10.1186/s13063-022-06971-w)
Supplement: Supplementary file 3 — Additional file 3. LongSTEP Treatment Delivery Tools. [file 13063_2022_6971_MOESM3_ESM.pdf]

LongSTEP Treatment Delivery Tool A

NICU phase, external rater version

Rater initials:
Site:
Therapist initials:
OC Participant ID:
Session number:

While listening to the audio recording of <u>*this specific*</u> *music therapy session, please rate how much you agree with the statements below. The box all the way to the left means, "I completely disagree" with the statement, while the box all the way to the right means, "I completely agree" with the statement. Other* answers *can be marked in the boxes in between.*

1. In the beginning of the session, music therapist and parent(s) observe and dialogue on infant's current state and needs prior to presenting any music

I completely disagree								I completely agree

2. In the beginning of the session (and during, as appropriate), music therapist dialogues with parent(s) about parent's own state, needs, and concerns

I completely disagree								I completely agree

3. Voice serves as the main instrument during the music therapy session

I completely disagree								I completely agree

4. Parental voice serves as a prominent musical voice during the music therapy session

I completely disagree								I completely agree

5. Music therapist provides opportunities for parents to actively participate during the music therapy session

I completely disagree								I completely agree

6. Music is modified in response to infant's cues and responses during the music therapy session

I completely disagree								I completely agree

Examples of discrete behaviors to look for

1. **In the beginning of the session, music therapist and parent(s) are observing and dialoguing on infant's current state and needs prior to presenting any music**
   Music therapist and parents observe and talk together about infant's state in the moment, physical positioning, infant strengths/resources and challenges that parents have observed lately e.g. development in self-regulatory capacities/strategies, tolerance to sensory input (e.g. touch, handling, noise etc.) communication/interaction (eye-contact, smiles, alertness) and current medical or other challenges/concerns (e.g. feeding, coming off ventilation, pain etc.).

2. **In the beginning of the session (and during, as appropriate), music therapist dialogues with parent(s) about parent's own state, needs, and concerns**
   Music therapist invites parents to share how they are doing, as well as any thoughts, concerns, and questions they might have prior to the session, and during the session if appropriate (e.g. if a parent starts crying during the session, seems stressed).

3. **Voice serves as the main instrument during the music therapy session**
   Song and voice are the most evident sources of music offered. Other instruments (guitar, lyre) are used with moderation, preferably only when requested by parent or when considered necessary to promote/encourage continuation of parental singing (e.g. if parent is not comfortable with singing without support), and is delivered in an infant-appropriate way (e.g. simple accompaniment, appropriate tempo and volume).

4. **Parental voice serves a prominent musical voice during the music therapy session**
   Music therapist makes room for the parent to take on a leading role in singing. The parental voice is distinguishable during the music therapy session. When singing together with parents, the music therapist modifies volume of own singing and takes on a supportive role musically. If parents are hesitant or insecure about singing, music therapist demonstrates and supports parent in how spoken voice can be used in an infant-directed way with musical qualities (e.g. with variations in pitch, tempo, use of pauses, vocal inflexion etc.).

5. **Music therapist provides opportunities for parents to actively participate during the music therapy session**
   Music therapist encourages and guides parents to sing or hum in an infant-directed way, invites them to touch and move infant in response/relation to the music (when appropriate). If parents are resistant to singing, music therapist offers other ways of participating such as writing or modifying lyrics of parent-chosen songs. Music therapist creates adequate space for parents, asks questions, and enables parents to share experiences they have had with their baby so far (e.g. asking what strategies have been successful in soothing the child, how the baby prefers to be held, touched etc).

6. **Music is modified in response to infant cues and responses throughout the music therapy session**
   Song and voice use within the session seems to be infant-directed, and attuned to infant cues and responsivity. Parent(s) and music therapist is observed to attend to and modify music according to infant's state (e.g. changes in alertness, responsivity) engagement/ disengagement cues, and pauses/stops music in response to signs of overstimulation.

LongSTEP Treatment Delivery Tool B

NICU phase, music therapist self-rater version

Site:
Therapist initials:
OC Participant ID:
Session number:

While listening to the audio recording of this specific music therapy session, please rate how much you agree with the statements below. The box all the way to the left means, "I completely disagree" with the statement, while the box all the way to the right means, "I completely agree" with the statement. Other answers *can be marked in the boxes in between.*

1. **In the beginning of the session, music therapist and parent(s) observe and dialogue on infant's current state and needs prior to presenting any music**

I completely disagree								I completely agree

2. **In the beginning of the session (and during, as appropriate), music therapist dialogues with parent(s) about parent's own state, needs, and concerns**

I completely disagree								I completely agree

3. **Voice serves as the main instrument during the music therapy session**

I completely disagree								I completely agree

4. **Parental voice serves as a prominent musical voice during the music therapy session**

I completely disagree								I completely agree

5. **Music therapist provides opportunities for parents to actively participate during the music therapy session**

I completely disagree								I completely agree

6. **Music is modified in response to infant's cues and responses during the music therapy session**

I completely disagree								I completely agree

7. **Parents' musical preferences and abilities are integrated and accommodated into the music therapy session**

I completely disagree								I completely agree

Examples of discrete behaviors to look for

1. **In the beginning of the session, music therapist and parent(s) are observing and dialoguing on infant's current state and needs prior to presenting any music**
   Music therapist and parents observe and talk together about infant's state in the moment, physical positioning, infant strengths/resources and challenges that parents have observed lately e.g. development in self-regulatory capacities/strategies, tolerance to sensory input (e.g. touch, handling, noise etc.) communication/interaction (eye-contact, smiles, alertness) and current medical or other challenges/concerns (e.g. feeding, coming off ventilation, pain etc.)**.**

2. **In the beginning of the session (and during, as appropriate), music therapist dialogues with parent(s) about parent's own state, needs, and concerns**
   Music therapist invites parents to share how they are doing, as well as any thoughts, concerns, and questions they might have prior to the session, and during the session if appropriate (e.g. if a parent starts crying during the session, seems stressed).

3. **Voice serves as the main instrument during the music therapy session**
   Song and voice are the most evident sources of music offered. Other instruments (guitar, lyre) are used with moderation, preferably only when requested by parent or when considered necessary to promote/encourage continuation of parental singing (e.g. if parent is not comfortable with singing without support), and is delivered in an infant-appropriate way (e.g. simple accompaniment, appropriate tempo and volume).

4. **Parental voice serves a prominent musical voice during the music therapy session**
   Music therapist makes room for the parent to take on a leading role in singing. The parental voice is distinguishable during the music therapy session. When singing together with parents, the music therapist modifies volume of own singing and takes on a supportive role musically. If parents are hesitant or insecure about singing, music therapist demonstrates and supports parent in how spoken voice can be used in an infant-directed way with musical qualities (e.g. with variations in pitch, tempo, use of pauses, vocal inflexion etc.).

5. **Music therapist provides opportunities for parents to actively participate during the music therapy session**
   Music therapist encourages and guides parents to sing or hum in an infant-directed way, invites them to touch and move infant in response/relation to the music (when appropriate). If parents are resistant to singing, music therapist offers other ways of participating such as writing or modifying lyrics of parent-chosen songs. Music therapist creates adequate space for parents, asks questions, and enables parents to share experiences they have had with their baby so far (e.g. asking what strategies have been successful in soothing the child, how the baby prefers to be held, touched etc).

6. **Music is modified in response to infant cues and responses throughout the music therapy session**
   Song and voice use within the session seems to be infant-directed, and attuned to infant cues and responsivity. Parent(s) and music therapist is observed to attend to and modify music according to infant's state (e.g. changes in alertness, responsivity) engagement/disengagement cues, and pauses/stops music in response to signs of overstimulation.

7. **Parents' musical preferences and abilities are integrated into the music therapy session** Parents are encouraged to make share preferences and familiar songs that in turn, are integrated and accommodated within the session in adapted forms. "Preferred songs" refers to both lullabies and other types of music (e.g. pop music, music representing parents' nationality/culture/religion/beliefs etc.). Music therapist accommodates to parent's musical abilities (e.g. no/some experience with singing) and modifies music (e.g. models/accompanies in comfortable vocal range/key) to facilitate musical engagement of the parent. The importance of the parental voice for the infant is emphasized, and spontaneous infant-directed speech, and vocalizations from the parent are encouraged/welcomed.

LongSTEP Treatment Delivery Tool C

Post-discharge phase, external rater version

Rater initials:
Site:
Music therapist initials:
OC Participant ID:
Session number:

Please specify who are visible in the video frame and your access to observe facial expressions and body movements

Fully visible= *Facial expressions and body movements easily observed*
Partly visible= *Facial expressions and body movements somewhat possible to observe*
Limited visibility= *Difficult or not possible to see facial expressions, and limited view of body movements*
Not visible, but audible= *Present and audible, but not visible*
Not visible, nor audible *= Present, but neither visible nor audible*

*Place an "**X**" in the applicable cell for each participant present in the session*

	Infant	Parent 1 (mother)	Parent 2 (partner, father)	Music therapist	Other (specify)
Not present					
Fully visible					
Partly visible					
Limited visibility					
Not visible, but audible					
Not visible, nor audible					

While watching the video recording of this specific music therapy session, please rate how much you agree with the statements below. The box all the way to the left means, “I completely disagree” with the statement, while the box all the way to the right means, “I completely agree” with the statement. Other answers *can be marked in the boxes in between.*

1. **In the beginning of the session, music therapist and parent(s) observe and dialogue on infant’s current state and needs prior to presenting any music**

I completely disagree								I completely agree

2. **In the beginning of the session (and during, as appropriate), music therapist dialogues with parent(s) about parent’s own state, needs, and concerns**

I completely disagree								I completely agree

3. **In the beginning of the session (and during, as appropriate), infant’s physical positioning is attended to and adjusted to support infant self-regulation and enhance conditions for interaction between infant and parent(s)**

I completely disagree								I completely agree

4. **Voice serves as the main instrument during the music therapy session**

I completely disagree								I completely agree

5. **Parental voice serves as a prominent musical voice during the music therapy session**

I completely disagree								I completely agree

6. **Music therapist provides opportunities for parents to actively participate during the music therapy session**

I completely disagree								I completely agree

7. **Music is modified in response to infant’s cues and responses during the music therapy session**

I completely disagree								I completely agree

Examples of discrete behaviors to look for

1. **In the beginning of the session, music therapist and parent(s) are observing and dialoguing on infant's current state and needs prior to presenting any music**
   Music therapist and parents observe and talk together about infant's state in the moment, physical positioning, infant strengths/resources and challenges that parents have observed lately e.g. development in self-regulatory capacities/strategies, tolerance to sensory input (e.g. touch, handling, noise etc.) communication/interaction (eye-contact, smiles, alertness) and current medical or other challenges/concerns (e.g. feeding, coming off ventilation, pain etc.).

2. **In the beginning of the session (and during, as appropriate), infant's physical positioning is attended to and adjusted to support infant self-regulation and enhance conditions for interaction between infant and parent(s)**
   Music therapist and parent discuss how infant is currently positioned, as well as any infant preferences for positioning and containment that the parent is familiar with. If appropriate, music therapist may demonstrate containment strategies (e.g. static cupping of feet and top of head, static touch on infant's back, gathering of infant's hands, holding infant in slightly more upward position to promote alertness) for the parent to use during sessions.

3. **In the beginning of the session (and during, as appropriate), infant's physical positioning is attended to and adjusted to support infant self-regulation and enhance conditions for interaction between infant and parent(s)**
   Music therapist and parent discuss how infant is currently positioned, as well as any infant preferences for positioning and containment that the parent is familiar with. If appropriate, music therapist may demonstrate strategies to facilitate interaction for the parent to use during sessions (e.g. gathering of infant's hands, holding infant in slightly more upward position to promote alertness)

4. **Voice serves as the main instrument during the music therapy session**
   Song and voice are the most evident sources of music offered. Other instruments (guitar, lyre) are used with moderation, preferably only when requested by parent or when considered necessary to promote/encourage continuation of parental singing (e.g. if parent is not comfortable with singing without support), and is delivered in an infant-appropriate way (e.g. simple accompaniment, appropriate tempo and volume).

5. **Parental voice serves a prominent musical voice during the music therapy session**
   Music therapist makes room for the parent to take on a leading role in singing. The parental voice is distinguishable during the music therapy session. When singing together with parents, the music therapist modifies volume of own singing and takes on a supportive role musically.

6. **Music therapist provides opportunities for parents to actively participate during the music therapy session**
   Music therapist encourages and guides parents to sing or hum in an infant-directed way, invites them to touch and move infant in response/relation to the music (when appropriate). If parents are resistant to singing, music therapist offers other ways of participating such as writing or modifying lyrics of parent-chosen songs.

7. **Music is modified in response to infant cues and responses throughout the music therapy session**
   Song and voice use within the session seems to be infant-directed, and attuned to infant cues and responsivity. Parent(s) and music therapist is observed to attend to and modify music according to infant's state (e.g. changes in alertness, responsivity) engagement/disengagement cues, and pauses/stops music in response to signs of overstimulation.

LongSTEP Treatment Delivery Tool D

Post-discharge phase, self-rating version

Site:
Music therapist initials:
OC Participant ID:
Session number:

Please specify who are visible in the video frame and your access to observe facial expressions and body movements

Fully visible= *Facial expressions and body movements easily observed*
Partly visible= *Facial expressions and body movements somewhat possible to observe*
Limited visibility= *Difficult or not possible to see facial expressions, and limited view of body movements*
Not visible, but audible= *Present and audible, but not visible*
Not visible, nor audible *= Present, but neither visible nor audible*

*Place an **"X"** in the applicable cell for each participant present in the session*

	Infant	Parent 1 (mother)	Parent 2 (partner, father)	Music therapist	Other (specify)
Not present					
Fully visible					
Partly visible					
Limited visibility					
Not visible, but audible					
Not visible, nor audible					

While watching the video recording of this specific music therapy session, please rate how much you agree with the statements below. The box all the way to the left means, "I completely disagree" with the statement, while the box all the way to the right means, "I completely agree" with the statement. Other answers *can be marked in the boxes in between*

1. **In the beginning of the session, music therapist and parent(s) observe and dialogue on infant's current state and needs prior to presenting any music**

I completely disagree								I completely agree

2. **In the beginning of the session (and during, as appropriate), music therapist dialogues with parent(s) about parent's own state, needs, and concerns**

I completely disagree								I completely agree

3. **In the beginning of the session (and during, as appropriate), infant's physical positioning is attended to and adjusted to support infant self-regulation and enhance conditions for parent--infant interaction**

I completely disagree								I completely agree

4. **Voice serves as the main instrument during the music therapy session**

I completely disagree								I completely agree

5. **Parental voice serves as a prominent musical voice during the music therapy session**

I completely disagree								I completely agree

6. **Music therapist provides opportunities for parents to actively participate during the music therapy session**

I completely disagree								I completely agree

7. **Music is modified in response to infant's cues and responses during the music therapy session**

I completely disagree								I completely agree

8. **Parents' musical preferences and abilities are integrated and accommodated into the music therapy session**

I completely disagree								I completely agree

Examples of discrete behaviors to look for

1. In the beginning of the session, music therapist and parent(s) are observing and dialoguing on infant's current state and needs prior to presenting any music
Music therapist and parents observe and talk together about infant's state in the moment, physical positioning, infant strengths/resources and challenges that parents have observed lately e.g. development in self-regulatory capacities/strategies, tolerance to sensory input (e.g. touch, handling, noise etc.), communication /interaction (eye-contact, smiles, alertness) and current medical or other challenges/concerns (e.g. feeding, coming off ventilation, pain etc.)**.**

2. In the beginning of the session (and during, as appropriate), music therapist dialogues with parent(s) about parent's own state, needs, and concerns
Music therapist invites parents to share how they are doing, as well as any thoughts, concerns, and questions they might have prior to the session, and during the session if appropriate (e.g. if a parent starts crying during the session, seems stressed).

3. In the beginning of the session (and during, as appropriate), infant's physical positioning is attended to and adjusted to support infant self-regulation and enhance conditions for interaction between infant and parent(s)
Music therapist and parent discuss how infant is currently positioned, as well as any infant preferences for positioning and containment that the parent is familiar with. If appropriate, music therapist may demonstrate strategies to facilitate interaction for the parent to use during sessions (e.g. gathering of infant's hands, holding infant in slightly more upward position to promote alertness)

4. Voice serves as the main instrument during the music therapy session
Song and voice are the most evident sources of music offered. Other instruments (guitar, lyre) are used with moderation, preferably only when requested by parent or when considered necessary to promote/encourage continuation of parental singing (e.g. if parent is not comfortable with singing without support), and is delivered in an infant-appropriate way (e.g. simple accompaniment, appropriate tempo and volume).

5. Parental voice serves a prominent musical voice during the music therapy session
Music therapist makes room for the parent to take on a leading role in singing. The parental voice is distinguishable during the music therapy session. When singing together with parents, the music therapist modifies volume of own singing and takes on a supportive role musically.

6. Music therapist provides opportunities for parents to actively participate during the music therapy session
Music therapist encourages and guides parents to sing or hum in an infant-directed way, invites them to touch and move infant in response/relation to the music (when appropriate). If parents are resistant to singing, music therapist offers other ways of participating such as writing or modifying lyrics of parent-chosen songs. Music therapist creates adequate space for parents, asks questions, and enable parents to share experiences they have had with their baby so far (e.g. asking what strategies have been successful in soothing the child, how the baby prefers to be held, touched etc.)

7. Music is modified in response to infant cues and responses throughout the music therapy session
Song and voice use within the session seems to be infant-directed, and attuned to infant cues and responsivity. Parent(s) and music therapist is observed to attend to and modify music according to infant's state (e.g. changes in alertness, responsivity) engagement/disengagement cues, and pauses/stops music in response to signs of overstimulation.

8. Parents' musical preferences and abilities are integrated into the music therapy session Parents are encouraged to make share preferences and familiar songs that in turn, are integrated and accommodated within the session in adapted forms. "Preferred songs" refers to both lullabies and other types of music (e.g. pop music, music representing parents' nationality/culture/religion/beliefs etc.). Music therapist accommodates to parent's musical abilities (e.g. no/some experience with singing) and modifies music (e.g. models/accompanies in comfortable vocal range/key) to facilitate musical engagement of the parent. The importance of the parental voice for the infant is emphasized, and spontaneous infant-directed speech, and vocalizations from the parent are encouraged and welcomed.
