# Supplementary material for: Treatment fidelity in a pragmatic clinical trial of music therapy for premature infants and their parents: the LongSTEP study
Source: Trials. 2023 Mar 3;24:160. doi: 10.1186/s13063-022-06971-w (PMC9983212; doi:10.1186/s13063-022-06971-w)
Supplement: Supplementary file 4 — Additional file 4. LongSTEP Treatment Receipt Questionnaire. [file 13063_2022_6971_MOESM4_ESM.pdf]

E) LongSTEP Treatment Receipt Questionnaire

TR_1	Were you and your infant assigned to receive music therapy during this research study?	☐ Yes
		☐ No

If ***yes***, please continue to the questions below.
If ***no***, you are finished with this questionnaire. *Thank you for completing this questionnaire! We appreciate your time and effort*.

The following questions are for mothers who were assigned to receive music therapy as part of their participation in the study.

TR_2	When did you participate in music therapy during this research study? (*Please tick one box*).	☐	In the NICU
		☐	After being discharged from the NICU
		☐	Both in the NICU and after being discharged

Thinking back on your experiences with music therapy, please rate how much you agree with the statements below by marking an "X" in one of the boxes. The box all the way to the left means, "I completely agree" with the statement, while the box all the way to the right means, "I completely disagree" with the statement. Other answers can be marked in the boxes in between.

TR_3	Before the music started, the music therapist and I looked at my baby and discussed my baby's current state (for example asleep, awake, calm, distressed), and what the baby might need in the moment:

I completely agree								I completely disagree

TR_4	Before the music started, the music therapist and I checked if my baby was laying (or sitting) in a way that helped my baby be ready for music, and if needed we adjusted the baby's position:

I completely agree								I completely disagree

TR_5	Before the music started, the music therapist asked me about how I was feeling, and whether I had any particular concerns, as well as any wishes or needs for the music therapy session:

I completely agree								I completely disagree

TR_6	We used singing and our voices more than any other instrument during music therapy sessions:

I completely agree								I completely disagree

TR_7	The music therapist encouraged me to use my voice and singing to connect with my baby in the music therapy sessions:

I completely agree								I completely disagree

TR_8	I actively participated in the music therapy sessions. For example, by singing, humming, touching and/or moving my baby to the music, and/or making up songs and lyrics:

I completely agree								I completely disagree

TR_9	The music was adjusted according to my baby's reaction to it. For example, we changed tempo, volume, or melody to interact with my baby, or paused and made the music simpler if the baby seemed to need a break:

I completely agree								I completely disagree

TR_10	We used music that my family and I know and like in the music therapy sessions:

I completely agree								I completely disagree

TR_11	I experienced in the music therapy sessions that I have something unique and important to offer my baby through my voice:

I completely agree								I completely disagree

Thank you for completing this questionnaire! We appreciate your time and effort.
