# Supplementary material for: Treatment fidelity in a pragmatic clinical trial of music therapy for premature infants and their parents: the LongSTEP study
Source: Trials. 2023 Mar 3;24:160. doi: 10.1186/s13063-022-06971-w (PMC9983212; doi:10.1186/s13063-022-06971-w)
Supplement: Supplementary file 5 — Additional file 5: Table 4. Reliability for LongSTEP treatment fidelity questionnaires if an item is dropped. [file 13063_2022_6971_MOESM5_ESM.docx]

Table 4. Reliability for LongSTEP treatment fidelity questionnaires if an item is dropped

|  |  | Treatment delivery NICU | | |  | Treatment delivery PD | | |  | Treatment receipt  both phases |
| --- | --- | --- | --- | --- | --- | --- | --- | --- | --- | --- |
| α (CI) |  | MT self-rater |  | External rater |  | MT self-rater |  | External rater |  | Parent rater |
|  |  |  |  | |  |  |  |  |  |  |
| Item 1 |  | 0.74 (0.66, 0.82) | 0.65 (0.58, 0.72) | |  | 0.86 (0.82, 0.90) |  | 0.77 (0.73, 0.81) |  | 0.75 (0.69, 0.81) |
| Item 2 |  | 0.79 (0.73, 0.85) | 0.65 (0.57, 0.73) | |  | 0.88 (0.85, 0.91) |  | 0.75 (0.70, 0.80) |  | 0.75 (0.69, 0.81) |
| Item 3 |  | 0.70 (0.61, 0.79) | 0.60 (0.52, 0.68) | |  | 0.85 (0.81, 0.89) |  | 0.77 (0.73, 0.81) |  | 0.77 (0.72, 0.82) |
| Item 4 |  | 0.74 (0.66, 0.82) | 0.67 (0.60, 0.74) | |  | 0.85 (0.81, 0.89) |  | 0.78 (0.74, 0.82) |  | 0.78 (0.72, 0.84) |
| Item 5 |  | 0.66 (0.56, 0.76) | 0.56 (0.47, 0.65) | |  | 0.87 (0.83, 0.91) |  | 0.79 (0.75, 0.83) |  | 0.76 (0.70, 0.81) |
| Item 6 |  | 0.68 (0.59, 0.77) | 0.62 (0.54, 0.70) | |  | 0.84 (0.79, 0.88) |  | 0.77 (0.72, 0.82) |  | 0.79 (0.74, 0.84) |
| Item 7 |  | 0.69 (0.60, 0.78) | - | |  | 0.86 (0.82, 0.90) |  | 0.77 (0.72, 0.82) |  | 0.78 (0.73, 0.83) |
| Item 8 |  | - | - | |  | 0.85 (0.81, 0.89) |  | - |  | 0.76 (0.71, 0.81) |
| Item 9 |  | - | - | |  | - |  | - |  | 0.76 (0.71, 0.81) |
| Abbreviations: NICU, neonatal intensive care unit; CI, confidence interval; PD, post-discharge | | | | | | | | | | |
